# The impact of hemodiafiltration on cognitive function in patients with end-stage renal disease

**DOI:** 10.3389/fnins.2022.980658

**Published:** 2023-01-19

**Authors:** Xiaoyan Wang, Xiaohui Chen, Yuting Tang, Liuping Zhang, Yue Wang, Zhenghua Hou, Wenhao Jang, Yonggui Yuan

**Affiliations:** ^1^Department of Psychosomatics and Psychiatry, Zhongda Hospital, Medical School, Southeast University, Nanjing, Jiangsu, China; ^2^Department of Nursing, Zhongda Hospital, Medical School, Southeast University, Nanjing, Jiangsu, China; ^3^Department of Radiology, Zhongda Hospital, Medical School, Southeast University, Nanjing, Jiangsu, China; ^4^Institute of Nephrology, Zhongda Hospital, Medical School, Southeast University, Nanjing, Jiangsu, China

**Keywords:** end-stage renal disease, blood purification, hemodialysis, hemodiafiltration, cerebral blood flow, cognitive functions

## Abstract

**Background:**

Patients with end-stage renal disease are more likely to suffer cognitive impairment. Cognitive impairment may lead to long-term severe adverse consequences.

**Purpose:**

To explore the impact of different blood purification therapy on cerebral blood flow and cognitive functions in end-stage renal disease.

**Materials and methods:**

This prospective study evaluated patients with end-stage renal disease undergoing blood purification from January to March 2021. Matched healthy controls were also included. Participants performed neurocognitive measurements, including a mini-mental state examination, logical memory test-20-minutes delayed, verbal fluency test, digit span test, clock drawing test, and stroop color and word test C. In addition, we tested plasma amyloid-β protein levels, serum Fe and hemoglobin levels in blood samples. Cerebral blood flow was measured using pulsed pseudocontinuous arterial spin labeling. We analyzed and compared the correlation between cognitive function, biomarkers, and cerebral blood flow between patients and healthy subjects, as well as between patients with different treatments.

**Results:**

A total of 44 patients with end-stage renal disease (mean age, 57.39 years ± 8.63) and 46 healthy controls (mean age, 56.15 years ± 6.40) were recruited. Patients receive hemodialysis three times a week, and 27 of them have been replaced hemodialysis for hemodiafiltration twice a month. The cognitive function of patients was worse than healthy controls (*P* < 0.05). The patients showed higher plasma concentrations of amyloid-β40, amyloid-β42, Tau, and pTau181 than healthy controls (*P* < 0.05). The group receiving both hemodialysis and hemodiafiltration had higher cerebral blood flow signal values in the left caudate nucleus (chuster-level *P* < 0.05, voxel-level *P* < 0.001). They also exhibited better verbal fluency function than the hemodialysis-only group (*P* < 0.05).

**Conclusion:**

Patients with the end-stage renal disease showed widespread cognitive declines. Cerebral blood flow generally decreased in the cerebral cortex and increased in subcortical regions. The hemodiafiltration may protect verbal function by increasing cerebral blood flow in the left caudate.

## 1. Introduction

End-stage renal disease (ESRD) is severe chronic kidney disease. Extracorporeal blood purification (BP) therapies such as hemodialysis (HD) and hemodiafiltration (HDF) are the most widely used treatment for patients with ESRD. Patients with ESRD undergoing BP (ESRD-BP) are more common to occur cognitive impairment (CI) (Ali et al., 2021). CI may lead to long-term severe adverse consequences, including dementia and death (Angermann et al., 2018; McAdams-DeMarco et al., 2018).

The mechanism of CI in ESRD-BP is unclear. Uremic toxicity and cerebral neurovascular abnormalities are involved in the pathogenesis. Renal failure accumulates uremic toxins in the circulation, and the neurotoxicity of urinary toxins directly affects cognitive function. Protein-bound uremic toxins accumulate in plasma and systemic organs, such as indoxyl sulfate, and p-cresyl sulfate (Sato et al., 2018). Indoxyl sulfate induces neuroinflammation in glial cells, inflammation has been associated with neurodegenerative diseases such as CI (Adesso et al., 2018).

In contrast, elevated uremic toxins disrupt the blood-brain barrier (BBB) (Bobot et al., 2020; Viggiano et al., 2020). It affects the circulation of Alzheimer’s disease-related proteins such as amyloid-β (Aβ) and Tau proteins. HD effectively removes Aβ from the blood through the dialyzer (Kato et al., 2012). HDF, combined with the diffusion function of HD with convection, enhances macromolecule solute removal, including Aβ. However, the exact effect of HDF on Aβ dialysis and cognitive function remains unknown.

Though Aβ and Tau are an essential indicator of cognitive functions during BP, it is hard to detect the exact Aβ and Tau levels in the brain with acceptable spatial resolution. Notably, BP may pull the blood along with Aβ and Tau out for circulation, and the strength of this “pull” may be reflected by its impact on the cerebral blood flow (CBF) in distinct brain regions. Cerebrovascular damage in ESRD-BP patients, as well as BP-induced rapid changes in blood pressure, osmolarity, and acid-base balance, all of which affect the brain’s CBF. The percentage decrease in mean flow velocity was related to the decline in cognitive function, including overall function, executive function, and verbal fluency (Findlay et al., 2019). Pseudocontinuous arterial spin labeling (pCASL) has been shown to be a valid and safe method for assessment of regional CBF (Warnert et al., 2020). Here, we proposed to use pCASL to measure the whole brain and regional CBF as an indirect marker of Aβ and Tau in the brain space.

We hypothesized that HD and HDF would affect the CBF differently in patients with ESRD, leading to changes in blood flow that pull Aβ out in specific brain regions. We measured plasma Aβ, Tau protein levels, and CBF using pCASL. We aimed to perform case-control and BP-treated subgroups analyses on changes in regional CBF.

## 2. Materials and methods

This study was approved by the hospital ethics committee (approval number: 2020ZDSYLL294-P01) and registered with the Chinese Clinical Trial Registry (registration number: ChiCTR2100042710). All participants were recruited from the Department of Nephrology at hospital and informed and signed the consent forms before enrollment. The study was completed in accordance with the principles of the Declaration of Helsinki.

### 2.1. Participants

This prospective study evaluated patients and healthy controls (HCs) from January to March 2021. Patients with ESRD inclusion criteria were following: who met the ESRD diagnostic criteria and received HD three times a week for no less than 3 months, with or without HDF two times a month; were right-handed, aged 45–75 years, junior high school education or above; normal vision and hearing ability, normal limb muscle strength, no color weakness or blindness; could read and sign informed consent. Exclusion criteria included the following: patients with brain damage such as stroke or tumor; history of traumatic brain injury; with neuropsychiatric disorders; history of drug or alcohol abuse.

Then we matched the HCs by age, sex, and education level of the included ESRD-BP group. We recruited HCs from the local community in Nanjing through advertisements. Inclusion criteria of the HCs: who was healthy; mini-mental state examination (MMSE) score ≥25 points; were right-handed; no history of severe heart, brain, liver, or kidney disease; no history of alcohol and drug abuse; no family history of mental illness; read and sign informed consent. The PASS 11 software was used to calculate the sampling of each group according to the incidence of CI in ESRD and HCs. The sample size was estimated by comparing the correlation coefficients of the two samples with α = 0.05 (one-sided) and β = 0.10. The result was 21 cases. Assuming a loss to follow-up of 20%, we arrived at a sample size of at least 25 cases for each group.

### 2.2. Collection and analysis of blood samples

Blood samples were taken in the morning, before hemodialysis treatment. EDTA plasma was separated within 1 h after 3–5 ml blood was collected, and stored in a refrigerator at −80°. Using the fully automated SIMOA HD-X platform (GBIO, Hangzhou, China), the plasma Aβ40, Aβ42, Tau, and p-Tau181 concentrations were quantified using an ultra-sensitive Simoa^®^ technology (Quanterix, MA, US). The multiplex Neurology 3-Plex A kits (Cat. No. 101995) and pTau181-V2 assay kits (Cat. No.: 103714) were purchased from Quanterix. Calibrators and quality controls were measured in duplicate. All sample measurement was performed on a single run basis. Operators were unknown participants’ disease status.

### 2.3. Neuropsychological tests

All participants underwent the MMSE (Folstein et al., 1975), logical memory test-20-minutes delayed, verbal fluency test (VFT), digit span test, clock drawing test, stroop color and word test C. We computed compound scores in a fixed order to evaluate global cognition, memory, verbal fluency, executive function, visuospatial function.

### 2.4. MRI data acquisition

Using Philips MR Imaging DD 005 (Philips Ingenia II) MRI with a brain 16-channel coil to scaning in the study at the hospital. CBF was measured using pCASL. During the examination, the subjects were in a supine position, with a quiet and awake state, closed their eyes, and lay flat on the scanning bed. The subjects’ head and the coil were fixed with a foam pad to reduce head movement. We performed routine scans to exclude obvious intracranial lesions at first. Then we use pCASL technology to collect functional magnetic resonance imaging data of CBF. pCASL scan parameters: 3D-Grase sequence imaging, repetition time (TR) = 4464 ms, echo time (TE) = 12 ms, TI = 2000 ms, flip angle (FA) = 90°, acquisition matrix 64 × 60, field of view (FOV) = 240 mm × 240 mm, number of layers = 22 layers, thickness = 6 mm, layer spacing = 0 mm. Then use 3D-TFE sequence imaging technology to collect whole brain structure image data. T1-3D scan parameters: TR = 9.6 ms, TE = 3.7 ms, FA = 9°, acquisition matrix = 256 × 256, FOV = 256 mm × 256 mm, thickness = 1.1 mm, layer spacing = 0 mm.

### 2.5. MRI data processing and analysis

The improved perfusion data processing toolbox ASLtbx^1^ was used to calculate the CBF maps prior to spatial normalization (Wang et al., 2008). The cross point was set at the anterior commissure position for original images. After performing motion correction, the pCASL images were co-registered with the structural images and normalized to the Montreal Neurological Institute (MNI) space. We used the Gaussian kernel to smooth the pCASL to reduce noise. CBF maps were then calculated and normalized.

### 2.6. Statistical analysis

All data were processed using SPSS software (V20, IBM Corp., Armonk, NY, USA) and GraphPad Prism Version 8.0.1 (244). Kolmogorov–Smirnov was used for normality test for measurement data. Normal distribution data were reported as mean ± SD, *t*-test, or Pearson correlation analysis was used. Non-normal distribution data were reported as median (25% digits, 75% digits), non-parametric *t*-test was used to compare groups. Enumeration data were reported by frequency, and comparison between groups was performed by χ^2^ test. After D’Agostino-Pearson test Pearson’s correlation analysis was performed on laboratory data, CBF values and neuropsychological scale data. The difference was statistically significant with *P* < 0.05.

Multiple comparisons were corrected in normalized CBF maps comparison between groups were assessed with the Gaussian random field (GRF) at cluster-level *P* < 0.05 and voxel-level *P* < 0.001.

## 3. Results

**FIGURE 1:**
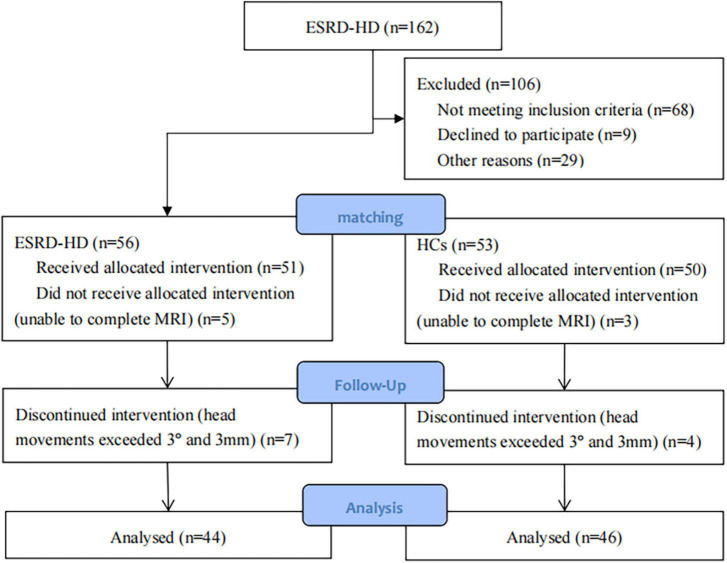
Flow diagram of sample selection. ESRD-HD, end-stage renal disease patients undergoing hemodialysis; HCs, healthy controls.

According to the inclusion criteria, we included 56 patients with ESRD-BP and 53 HCs. All participants received cognitive assessment, clinical examination, and MRI. Among them, 5 patients and 3 healthy controls were unable to complete MRI. Participants with head movements exceeding 3° and 3 mm (7 patients and 4 HCs) were excluded. Finally, participants who participated in the statistical analysis were 44 patients with ESRD-BP and 46 healthy subjects (Figure 1 and Table 1). There was no statistically significant difference between the two groups in age, sex, and education level (*P* > 0.05). There was a statistically significant difference between the two groups in MMSE (*P* < 0.05).

**TABLE 1:** Demographics between ESRD-BP and HCs groups.

Setting	ESRD-BP (*n* = 44)	HCs (*n* = 46)	*t*/*Z*/χ^2^ value	*P*-value
Age (in years)	57.39 ± 8.63	56.15 ± 6.40	0.768	0.445^a^
Education (in years)	9 (8, 11)	9 (8, 12)	−0.131	0.895^b^
Sex (M/F)	25/19	25/21	0.056	0.814^c^
Smoking (Y/N)	25/19	16/30	4.403	0.036^c^
Drinking (Y/N)	14/30	12/34	0.360	0.549^c^
eGFR (ml/min/1.73 m^2^)	7.5 (5, 14)	103 (99, 109)	−8.177	<0.001^b^
BMI (kg/m^2^)	23.19 ± 4.04	23.44 ± 2.32	−0.357	0.722^a^
HD duration (in months)	81.68 ± 43.126	N/A	N/A	N/A
HDF (Y/N)	28/16	N/A	N/A	N/A
MMSE/30 (score)	26 (23, 27)	28 (27, 29)	−4.384	<0.001^b^

^a^Independent two sample *t*-test;

^b^Mann–Whitney *U* test;

^c^χ^2^ test.

Non-normal distribution data were reported as median (25% digits, 75% digits). BMI, body mass index; eGFR, estimated glomerular filtration rate; ESRD-BP, end-stage renal disease patients undergoing blood purification; F, female; HCs, healthy controls; HD, hemodialysis; HDF, hemodiafiltration; M, male; MMSE, mini-mental state examination; N, no; N/A, not applicable; Y, yes.

### 3.1. Neuropsychological assessments reduction rates between ESRD-BP and HCs groups

**FIGURE 2:**
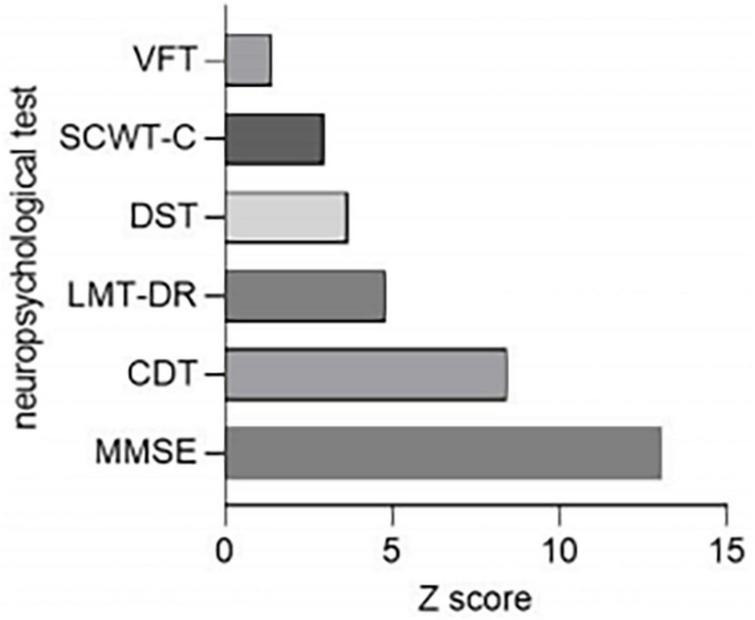
Comparison of neuropsychological tests reduction rates between ESRD-BP (*n* = 44) patients and HCs (*n* = 46). CDT, clock drawing test; DST, digit span test; ESRD-BP, end-stage renal disease patients undergoing blood purification; HCs, healthy controls; LMT-DR, logical memory test-20-minutes delayed; MMSE, mini-mental state examination; SCWT-C, stroop color and word test C; VFT, verbal fluency test.

The scores of MMSE, logical memory test-20-minutes delayed, VFT, digit span test, and clock drawing test in the ESRD-BP group were lower than those in the HCs group, and the stroop color and word test C score were higher than that in the HCs group. The result indicated that the cognitive function of the patient group was worse than the HCs group. Because the scale of the neurophysiological tests varied, the standardized Z-score was calculated to examine the deviations of each neuropsychological assessment (Z score = |X_ESRD–HD_-X_control_|/S_control_). Figure 2 showed that the overall cognitive changed the most, the verbal fluency changed the least.

### 3.2. Comparison of plasma protein concentrations between ESRD-BP and HCs groups

**FIGURE 3:**
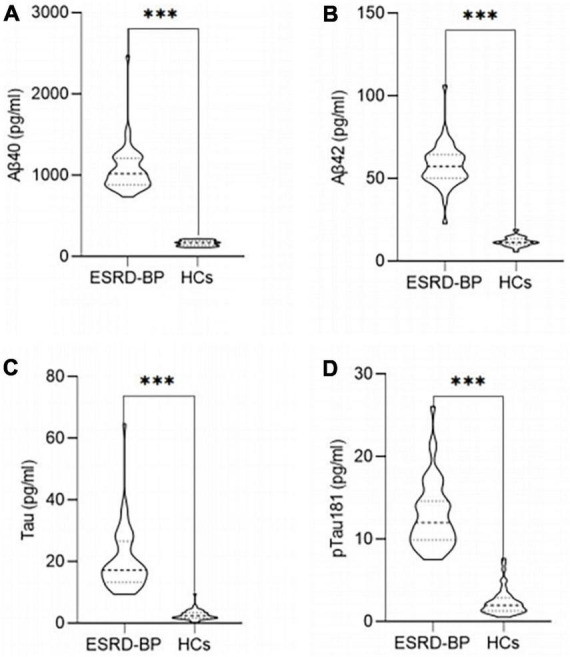
Comparison of the plasma concentrations of Aβ40 **(A)**, Aβ42 **(B)**, Tau **(C)**, and pTau181 **(D)** between ESRD-BP (*n* = 44) and HCs (*n* = 46) (^***^P < 0.05). ESRD-BP, end-stage renal disease patients undergoing blood purification; HCs, healthy controls.

The plasma concentrations of Aβ40, Aβ42, Tau, and pTau181 in the peripheral blood of the ESRD-BP group were higher than those of the HCs (Figures 3A–D). The difference between the two groups was statistically significant (*P* < 0.05).

### 3.3. Comparison of demographic information, biological indicators, neurocognitive evaluation between HD + HDF and HD groups

Patients with regular HD three times a week were allocated into two groups according to whether they were accompanied by HDF twice a month. We compared the age, sex, education level, eGFR, HD duration, whether with high blood pressure history, whether with diabetes history, blood pressure and blood glucose levels, peripheral blood serum Fe content and hemoglobin (Hb) content, and plasma Aβ40, Aβ42, Tau, pTau181 concentration, Aβ42/Aβ40 ratio, neurocognitive assessments such as MMSE, logical memory test-20-minutes delayed, VFT, digit span test, clock drawing test, stroop color, and word test C. Among them, the Aβ42/Aβ40, blood glucose levels, and VFT score difference between the two groups is statistically significant (Table 2).

**TABLE 2:** Demographics, biochemical parameters, and neuropsychological tests of patients with ESRD-BP.

Characteristic	HD + HDF (*n* = 27)	HD (*n* = 17)	*t*/χ^2^/*Z*-value	*P*-value
Age (years)	55.67 ± 8.199	60.12 ± 8.831	−1.702	0.096^a^
Sex (male/female)	14/13	11/6	0.703	0.402^b^
Education (years)	11 (8, 11.5)	9 (8, 11)	−1.038	0.299^c^
eGFR (ml/min/1.73 m^2^)	7 (5, 14)	8 (6, 12)	−0.146	0.884^c^
HD/M	90.00 ± 44.784	68.47 ± 37.908	1.644	0.108^a^
Hypertension (Y/N)	26/1	16/1	0.114	0.736^b^
Diabetes (Y/N)	7/20	8/9	2.073	0.150^b^
Systolic pressure (mmHg)	133.11 ± 13.169	137.47 ± 22.023	−0.737	0.468^a^
Diastolic pressure (mmHg)	75.93 ± 9.655	73.53 ± 10.393	0.778	0.441^a^
Blood glucose (mmol/L)	5.52 (4.71, 7.69)	8.78 (5.31, 13.00)	−2.302	0.021^b^
Fe (mmol/L)	46 (27.3, 88.9)	31.2 (10.1, 50)	−1.796	0.073^c^
Hb (g/L)	105.00 ± 16.399	114.76 ± 16.551	−1.916	0.062^a^
Aβ40 (pg/mL)	1020.68 (886.39, 1187.62)	1020.83 (941.08, 1245.76)	−0.349	0.727^c^
Aβ42 (pg/mL)	60.78 ± 14.482	53.52 ± 8.934	1.853	0.071^a^
Tau (pg/mL)	18.14 (12.48, 26.57)	16.95 (14.74, 20.79)	−0.229	0.819^c^
pTau181 (pg/mL)	12.57 ± 3.324	13.44 ± 5.006	−0.693	0.492^a^
Aβ42/Aβ40	0.06 ± 0.012	0.05 ± 0.007	2.086	0.043^a^
MMSE/30 (score)	27 (24, 28.5)	24 (22, 27)	−1.900	0.057^c^
LMT-DR/20 (score)	3 (2, 4)	3 (0, 5)	−0.341	0.733^c^
VFT (score)	12.96 ± 3.907	9.47 ± 2.896	3.172	0.003^a^
DST/20 (score)	11.15 ± 2.522	9.59 ± 2.874	1.893	0.065^a^
CDT/10 (score)	9 (6, 10)	8 (6, 9)	−1.116	0.264^c^
SCWT-C (second)	90 (72, 121.5)	98 (82, 125)	−0.410	0.682^c^

^a^Independent two sample *t*-test;

^b^χ^2^ test;

^c^Mann–Whitney *U* test.

Non-normal distribution data were reported as median (25% digits, 75% digits). BMI, body mass index; eGFR, estimated glomerular filtration rate; ESRD-BP, among end-stage renal disease patients undergoing blood purification; F, female; HCs, healthy controls; HD, hemodialysis; HDF, hemodiafiltration; LMT-DR, logical memory test-20-minutes delayed; M, male; MMSE, mini-mental state examination; m, month; N, no; SCWT-C, stroop color and word test C; VFT, verbal fluency test; DST, digit span test; CDT, clock drawing test; y, year; Y, yes.

### 3.4. Different brain regions of CBF between HD + HDF and HD groups

**FIGURE 4:**
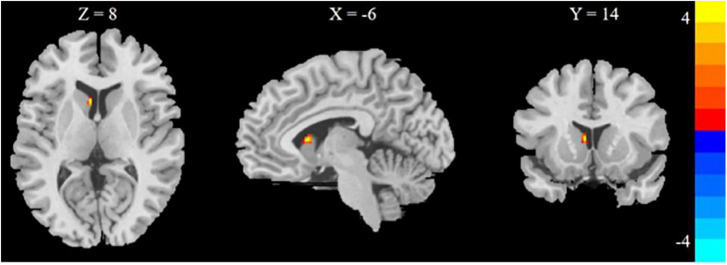
Regions with different brain regions with voxel-wise group comparison of cerebral blood flow in the HD + HDF group compared to HD group (cluster-level *P* < 0.05, voxel-level *P* < 0.001, GRF correction). Color bar indicates *t* scores. x, y, z = coordinates in Montreal Neurological Institute space. HD, hemodialysis; HDF, hemodiafiltration.

Comparison in the HD + HDF and HD groups of brain regions showed that higher CBF value of the left caudate nucleus in the HD + HDF group was increased than HD group (Table 3 and Figure 4).

**TABLE 3:** Comparisons of CBF in different brain regions between HD + HDF (*n* = 27) and HD (*n* = 17).

Brain regions (AAL)	MNI atlas coordinates			Voxels	Peak *t*-value
	**X**	**Y**	**Z**		
Caudate_L	−6	14	8	26	4.2006

AAL, anatomical automatic labeling; Caudate_L, left Caudate; HD, hemodialysis; HDF, hemodiafiltration; L, left; MNI, Montreal Neurological Institute. The significance threshold was set at *P* < 0.01 corrected for multiple comparisons with GRF correction at the voxel level.

### 3.5. Correlation of Aβ42/Aβ40, blood glucose levels, and the CBF values of left caudate nucleus with VFT scores in ESRD

**FIGURE 5:**
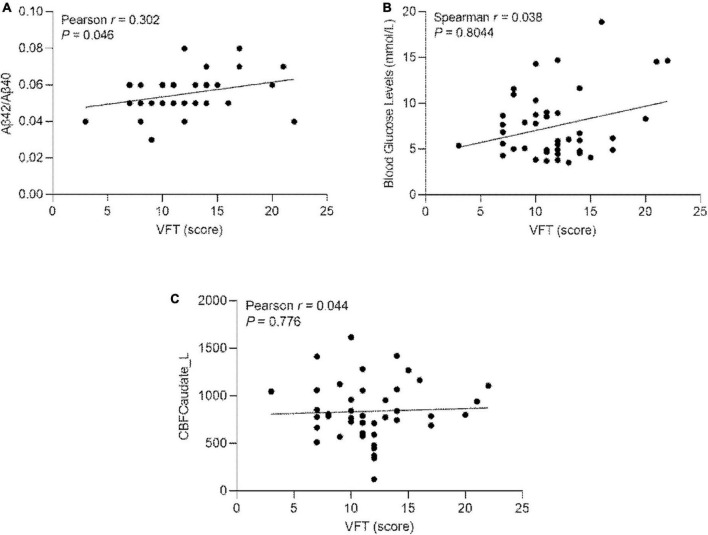
**(A)** Pearson’s correlation of the Aβ42/Aβ40 ratio and the VFT scores in ESRD (*n* = 44); **(B)** Pearson’s correlation of blood glucose levels and VFT scores in ESRD (*n* = 44). **(C)** Pearson’s correlation of the CBF values of left caudate nucleus and the VFT scores in ESRD (*n* = 44). Caudate_L, left Caudate; CBF, cerebral blood flow; VFT, verbal fluency test.

The Aβ42/Aβ40 ratio, blood glucose levels, the CBF values of left caudate nucleus and VFT score were all passed the D’Agostino-Pearson test. There is correlation between Aβ42/Aβ40 ratio and VFT scores in ESRD (*r* = 0.302; *P* = 0.046) (Figure 5A). There is no correlation between blood glucose levels and VFT scores in ESRD (*r* = 0.038; *P* = 0.804) (Figure 5B). There is no correlation between the CBF value of the left caudate nucleus and VFT scores in ESRD (*r* = 0.044; *P* = 0.776) (Figure 5C).

## 4. Discussion

We assessed cognitive functions and the cognition-related peripheral plasma proteins in patients with end-stage renal disease on long-term regular dialysis. We discovered that the cognitive function of the patients was worse than the healthy controls. The largest difference between groups was in overall cognitive function, while the verbal fluency suffered the least. The plasma concentrations of amyloid-β40, amyloid-β42, Tau, and pTau181 of the end-stage renal disease group were higher than the healthy controls.

We further divided the patients into two subgroups according to whether HDF was used. The results showed that the HDF group had higher plasma Aβ42/Aβ40 ratio and better cognitive function. We also found that ESRD patients with a higher Aβ42/Aβ40 ratio might have better verbal fluency. These findings could be, in part, attributed to HDF can dialyze more Aβ40 and less Aβ42 than HD. In addition, Aβ40 was more closely associated with CI than Aβ42 in AD (Yang et al., 2020). Existing evidence shows that the plasma Aβ42/Aβ40 ratio can predict Aβ-PET positivity (Pérez-Grijalba et al., 2019). The water-soluble Aβ was present in monomers and oligomers ranging from less than 10–100 kDa. Aβ40 and Aβ42 are the most abundant isoforms in humans (Mori et al., 1992). Blood-derived Aβ can enter the brain and induce neuronal dysfunction (Bu et al., 2018). At the same time, HD removes soluble Aβ from the blood while reducing senile plaques (Sakai et al., 2016). It was found that the uremic toxin indole sulfate-induced activation of aryl hydrocarbon receptor (AhR) may lead to disruption of the BBB in non-chronic kidney disease AhR^–/–^ knockout mice (Bobot et al., 2020). Disrupted BBB in patients with chronic kidney disease may increase BBB permeability. Researchers have also proposed that soluble human Aβ40 monomers can diffuse across the paracellular pathway of the BBB in a murine model of AD from the brain to the blood, and cognitive function was enhanced (Keaney et al., 2015). Long-term HD can promote the outflow of Aβ from the brain to the peripheral blood through the BBB, helping to maintain cognitive function (Kitaguchi et al., 2015).

Blood purification therapy removes waste products or toxins from the blood circulation by convection and diffusion treatments. HDF is a special method of blood purification. It based on HD using a highly permeable dialysis filtration membrane to increase the ultrafiltration rate and filter out large amounts of toxin-containing body fluids from the blood, while inputting an equal amount of replacement fluid. HDF is indicated for patients with intractable hypertension, hemodynamic instability, and intolerance to dialysis. HD is the efflux of toxins into the outflow hemodialysate fluid by convection, removing “small molecules” (0.5 kDa). HDF is an extracorporeal renal-replacement technique in which diffusion and convection are conveniently combined to remove “middle molecules” (0.5–5 kDa). Therefore, HDF treatment may remove more Aβ40, which accounts for 90% of the total Aβ, from human peripheral blood, resulting in a higher Aβ42/Aβ40 ratio, and is associated with better verbal fluency function. The general pros of HDF are that it improves the quality of the patient’s dialysis, makes the function of the cardiovascular system more stable, ensures the quality of the patient’s survival, and reduces cardiovascular mortality (Locatelli et al., 2018; Vernooij et al., 2022). The general cons of HDF is that it removes a portion of the nutrients from the patient’s body at the same time, which means that it leads to nutrient loss, and therefore should not be used as a routine form of dialysis.

Additionally, pCASL was used to measure the CBF in patients with ESRD-BP, and we expected to use it as an indirect indicator for Aβ. HDF adds convection to the diffusion of HD, and when blood is filtered, transmembrane pressure is formed, so the effect on CBF varies. We performed a CBF-based brain map comparison between the two subgroups. The HDF group showed higher CBF of the left caudate nucleus than the HD group. Brain perfusion levels are associated with cognitive function. A prospective longitudinal study using arterial spin labeling imaging found a significant decrease in CBF after initiation of HD in older adults, suggesting that decreased cerebral perfusion may be related to the observed cognitive decline (Li et al., 2020).

Conversely, increased perfusion would benefit cognitive function. The caudate nucleus is located in the subcortical part of the basal ganglia, involved in executive function. The caudate nucleus is involved in phonologic and semantic verbal fluency (Chouiter et al., 2016). This study showed that the verbal fluency function by VFT was better in the HDF group. We found a correlation between Aβ42/Aβ40 ratio and VFT scores. However, we did not find a correlation between the CBF of the left caudate nucleus and VFT scores. The insignificant finding might be caused by the small sample size, indicating that changes in the plasma Aβ42/Aβ40 ratio have an earlier and more significant effect on cognition function than changes in CBF of brain regions. The treatment modality for HDF may protect verbal fluency in ESRD patients by increasing blood perfusion in the left caudate nucleus.

Our study had some limitations. First, the lack of associations between CBF of the left caudate nucleus and neurocognitive assessment may reflect a power issue. Second, we compared two hemodialysis modalities, HDF and HD, but did not include patients undergoing peritoneal dialysis. Finally, the exact mechanism of how HDF increases CBF was not studied. In the future, multi-center and large-sample studies are needed to explore how the mechanism of HDF affects cognition function.

In summary, the hemodiafiltration group showed better verbal fluency function among patients with end-stage renal disease and a higher amyloid-β42/amyloid-β40 ratio than the hemodialysis group. The increased cerebral blood perfusion might protect the cognitive function.

## Data availability statement

The original contributions presented in this study are included in the article/supplementary material, further inquiries can be directed to the corresponding authors.

## Ethics statement

The studies involving human participants were reviewed and approved by IEC for Clinical Research of Zhongda Hospital, Affiliated to Southeast University. The patients/participants provided their written informed consent to participate in this study.

## Author contributions

XW, XC, LZ, and YW: guarantors of integrity of entire study. XW, XC, YT, LZ, YW, ZH, WJ, and YY: study concepts/study design or data acquisition or data analysis/interpretation, manuscript drafting or manuscript revision for important intellectual content, approval of final version of submitted manuscript, and agrees to ensure any questions related to the work are appropriately resolved. XW and YT: literature research and statistical analysis. XW, XC, LZ, YW, and ZH: clinical studies. XW: writing—original draft. WJ and YY: writing—review and editing. All authors contributed to the article and approved the submitted version.

## Footnotes

1
http://cfn.upenn.edu/∼zewang
